# Choroidal Changes in Blood Flow in Patients with Intermediate AMD after Oral Dietary Supplement Based on Astaxanthin, Bromelain, Vitamin D3, Folic Acid, Lutein, and Antioxidants

**DOI:** 10.3390/medicina58081092

**Published:** 2022-08-12

**Authors:** Rossella D’Aloisio, Luca Di Antonio, Lisa Toto, Marco Rispoli, Angelo Di Iorio, Giancarlo Delvecchio, Rodolfo Mastropasqua

**Affiliations:** 1 Department of Medicine and Science of Ageing, Ophthalmology Clinic, Chieti-Pescara, 66100 Chieti, Italy; 2 Ophthalmology Department UOSD Medical Retina, Eye Hospital, 00193 Rome, Italy; 3 Department of Innovative Technologies in Medicine & Dentistry, University “G. d’Annunzio”; Chieti-Pescara, 66100 Chieti, Italy; 4 Department of Ophthalmology, University of Modena and Reggio Emilia, 41121 Modena, Italy

**Keywords:** astaxanthin, choroidal blood flow, intermediate AMD, optical coherence tomography angiography

## Abstract

*Background and Objectives*: The aim of this study was to investigate the impact of oral administration of the combination of astaxanthin (AXT), lutein, folic acid, vitamin D3, and bromelain with antioxidants on choroidal blood flow in patients with age-related intermediate macular degeneration (AMD). *Materials and Methods*: Patients affected by intermediate AMD and treated with daily oral nutritional supplement with AXT, bromelain, vitamin D3, folic acid, lutein, and antioxidants for a period of at least 6 months were included in this retrospective study. A control group homogenous for age and sex was also included in the analysis. All participants underwent a complete ophthalmologic examination, spectral domain optical coherence tomography (SD-OCT), and optical coherence tomography angiography (OCTA) evaluation. Outcome measures were choroidal thickness (CHT) and choriocapillary vessel density (CCVD) after six months of AXT assumption. *Results*: CCVD values showed statistically significant difference between cases and controls at baseline (*p* < 0.001) and in the cases during follow-up (*p* < 0.001). The CHT measurements showed statistically significant difference between cases and controls (*p* = 0.002) and in the cases during follow-up (*p* < 0.001). *Conclusions*: The combined use of structural OCT and OCTA allows for a detailed analysis in vivo of perfusion parameters of the choriocapillaris and choroid and evaluation of changes of choroidal blood flow after oral nutritional supplements that affect blood flow velocity.

## 1. Background

Age-related macular degeneration (AMD) is a leading cause of central vision loss among people over age 55 years in developed countries [1].

Two main subtypes of AMD have been clinically recognized: neovascular or wet AMD, and non-neovascular or dry AMD [2]. The latter accounts for about 90% of all cases of AMD [3].

In its early stages, dry AMD is characterized by the presence of drusen (focal deposits of extracellular materials that are the hallmarks of dry AMD) and pigmentary abnormalities resulting from alterations in the retinal pigment epithelium (RPE) and Bruch’s membrane (BM). In late stages, dry AMD usually can progress to geographic atrophy (GA) associated to outer retinal degeneration and loss of photoreceptors and choriocapillaris [4]. Several factors have been implicated in AMD pathogenesis such as ageing, genetic predisposition, environmental elements, inflammation, and oxidative damage [5,6]. Different studies have shown that vascular changes may play a key role in AMD pathogenesis [7]. In particular, it has been pointed out as retinal and choroidal blood flows are reduced in AMD [7,8,9]. The latter aspect causing hypoxia is associated with the development of the drusen and with the progression of AMD towards to late pathways: neovascularization or atrophy [10]. A treatment regimen with astaxanthin (AXT), the most common carotenoid showing significant antioxidant properties, has been designed to elevate the choroidal blood flow velocity, and it should be of benefit in patients with AMD by improving the choroidal microcirculation [11,12]. Recently, the optical coherence tomography angiography (OCTA) has been introduced into daily clinical practice as a fast and safe method for imaging retinal and choroidal microcirculation by revealing the features that usually are not visualized with other imaging techniques [13,14,15]. The advent of OCTA allowed for visualizing the vascular changes that occur in dry AMD [16]. Particularly, it was suggested that eyes with dry AMD had a general impairment of choriocapillaris density compared to age-matched normal controls [16]. The aim of this study was to investigate the effect of oral administration of AXT together with bromelain, folic acid, vitamin D3, and antioxidants on choroidal blood flow in patients with intermediate AMD.

## 2. Methods

We retrospectively reviewed the electronic charts of 15 consecutive patients affected by intermediate AMD treated with daily oral nutritional supplement with AXT, antioxidants, lutein, bromelain, folic acid and D_3_ vitamin (Astazin 10^®^) for a period of at least 6 months.

All patients who consecutively presented to the Ophthalmology Department of the University “G. d’Annunzio” Chieti-Pescara Italy were recruited.

**Figure 1:**
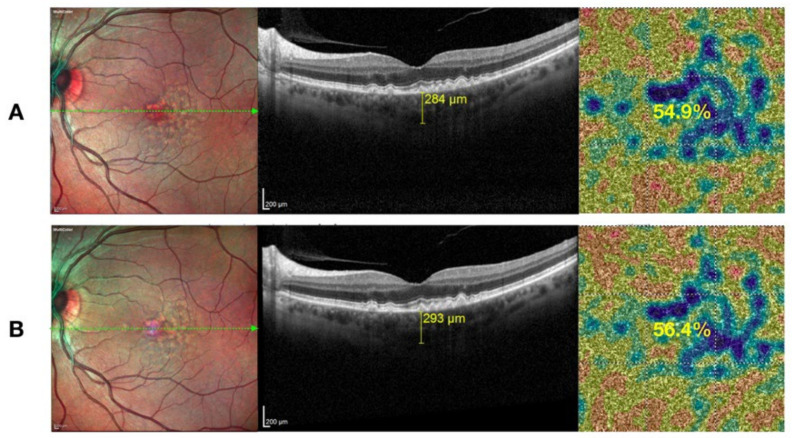
Multimodal retinal imaging of a 69-year-old woman affected by intermediate AMD at baseline and during follow-up. Multicolor imaging showing the presence of drusen and slight pigmentary abnormalities without clinical change at baseline (Panel (**A**), left) and after six months (Panel (**B**), left). SD-OCT scans showing typical drusen as focal deposits of extracellular materials between RPE and BM and choroidal thickness (CHT) measurement changes during the follow-up. Note the improvement of CHT value at baseline (Panel (**A**), middle) and after six months (Panel (**B**), middle). OCTA showing the color-coded flow density map of the choriocapillary vessel density (CCVD) changes during the follow up (the warmer the color, the greater the flow). Note the improvement of CCVD value at baseline (Panel (**A**), right) and after six months (Panel (**B**), right).

The study adhered to the tenets of the Declaration of Helsinki. Informed consent was obtained before the enrollment. The staging of AMD was based on the clinical AMD classification proposed by Ferris et al. [4]. Briefly, we considered only patients who presented large drusen (>125 μm) or with pigmentary abnormalities associated with at least medium drusen assessed by means of spectral-domain optical coherence tomography (SD-OCT), with no history of diabetes mellitus or arterial hypertension. Criteria for inclusion were age > 50 years old, best-corrected visual acuity (BCVA) of at least 0.1 logarithm of the minimum angle of resolution (LogMAR). Exclusion criteria were the presence of choroidal neovascularization or geographic atrophy, previous ocular surgery, any maculopathy secondary to causes other than AMD (including presence of an epiretinal membrane or vitreomacular traction syndrome), and significant media opacities. Each subject underwent a comprehensive ophthalmic examination, including BCVA using an Early Treatment Diabetic Retinopathy Study charts, slit-lamp biomicroscopy, intraocular pressure measurement with Goldman applanation tonometry, dilated funduscopic examination using a 78D (diopters) lens, a comprehensive multimodal retinal imaging procedure based on multicolor (MC) retinal images and SD-OCT using a confocal scanning laser ophthalmoscope (Spectralis HRA + OCT; Heidelberg Engineering, Heidelberg, Germany), and OCTA assessment by means of SD-OCTA (XR Avanti, Angiovue, Optovue Inc., Freemont, CA, USA) (Figure 1).

If both eyes met requirement criteria, one randomly selected eye of each subject was imaged.

**Figure 2:**
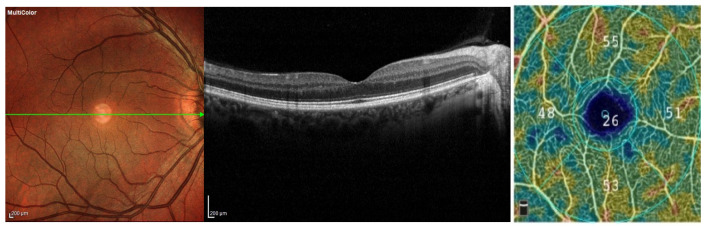
Multimodal retinal imaging of a healthy eye of 65-year-old. On OCTA scan foveal and parafoveal vessel density values are visible.

All patients affected by intermediate AMD had ingested one tablet a day after breakfast each composed by 12 mg/day of AXT (antioxidants, zinc, copper, lutein, bromelain and D_3_ vitamin) for a mean period of 180 ± 21 days. A control group (*n* = 13) homogenous for age and sex was also included in the analysis (Figure 2). All control subjects also underwent a complete ophthalmologic examination, including BCVA, intraocular pressure, fundus examination, and SD-OCT and OCTA evaluation.

Multicolor imaging and SD-OCT scan show a healthy eye without retinal abnormalities. OCTA shows the color-coded flow density map of the choriocapillary vessel density (CCVD).

SD-OCT and OCTA evaluation were performed in each patient at a time included between nine and eleven a.m.

Outcome measures included in this study were: choroidal thickness (CHT) and choriocapillaris vessel density (CCVD) after six months.

### Retinal Imaging Procedures

The MC retinal imaging was obtained using a commercially device (Spectralis HRA + OCT; Heidelberg Engineering, Heidelberg, Germany), which uses a confocal scanning laser ophthalmoscope to capture simultaneous reflectance images of the retina with three different wavelengths: blue (488 nm), green (515 nm), and infrared (828 nm) reflectances, respectively [17]. The MC images were captured with a scan covering a 30 × 30 degree macular field of view in order to enhance both drusen and pigmentary changes visualization.

The SD-OCT images were obtained with the high-speed resolution mode and Eye Tracking Automatic Real-Time System with a scan protocol covering a 30 × 30 degree macular cube area centered on the fovea. The CHT was manually measured in the sub-foveal location from the outer portion of the hyper-reflective band of the RPE to the inner surface of the sclera, and its value was calculated as the average of two manual measurement each taken by a single retinal specialist (L.D.A.). The OCTA assessment was performed by means of a commercially available device (XR Avanti, Angiovue, Optovue Inc, Freemont, CA, USA) that uses a scan speed of 70,000 A-scans per second and a light source centered on 840 nm, with a bandwidth of 45 nm. The OCTA images were processed using a split-spectrum amplitude decorrelation angiography (SSADA) algorithm (version 2018.1.0.24), providing axial and transverse resolutions of 15 microns in tissue for OCTA images. This algorithm identifies blood flow by calculating the decorrelation of signal amplitude from consecutive B-scans performed at the same retinal acquisition plane by splitting the spectrum, improving the signal-to-noise-ratio, and enhancing flow detection [17]. Study participants underwent OCTA imaging following a protocol that included two volumetric OCT dataset of 3 mm × 3 mm consisting of 304 × 304 pixels in the transverse dimension. Patients were instructed to fixate the target blue point during the image acquisition and avoid blinking or eye movements during the scanning process. After completion of the volumetric OCT dataset, the software applied motion-control technology to remove saccades and minor loss of fixation. Low quality scans (i.e., if the subject blinked or the scan had significant motion artifacts) were excluded and repeated until good quality scans were achieved; a signal strength index >60 was considered for the analysis. Two observers, independently, checked image quality and excluded poor quality images leading to possible segmentation errors. Eventual manual adjustment of layer segmentation in case of inaccurate segmentation was performed by two retina specialists (LDA and RDA), and the manual adjustment of segmentation was chosen only if a consensus was reached. For the quantitative analysis of choriocapillaris, a slab from BM–10μm to BM + 30μm was used [18], providing a consistent basis for the evaluation of the choriocapillaris across patients and over time. The CCVD measurement was calculated as a percentage of area occupied by vessels in a circular ROI centered on the center of the foveal avascular zone and with a diameter of 2.5 mm. AngioVue software automatically calculates the vessel density in the whole en-face (3 × 3 mm) angiogram as previously described [19].

## 3. Statistical Analysis

Data were reported as mean and standard deviation (SD). Differences between groups at baseline were tested with linear models for continuous variables, whereas chi-square test was used to test differences in categorical variables. To assess the variation in CHT and CCVD between baseline and follow-up, linear mixed models were applied only in the cases group, adjusted for age and sex.

Linear regression analysis with robust option was applied to evaluate correlations between variables of interest. Analyses were performed with the SAS statistical software, version 9.4. A two-sided *p*-value ≤ 0.05 was considered statistically significant.

## 4. Results

### 4.1. Demographic Data

Fifteen eyes (cases) from 15 patients (7 males and 8 females) with intermediate AMD and thirteen eyes (controls) from 13 healthy age-matched subjects (6 males and 7 females) were included in this study. The mean age was 70.20 ± 8.13 years in cases and 68.92 ± 8.62 years in controls (*p* = 0.70) (Table 1).

### 4.2. Functional and Morphological Results

BCVA showed no statistically significant difference between cases and controls (*p* = 0.34) (Table 1). The comparison between cases and control at baseline in CHT and CCVD showed statistically significant differences; moreover, an increase in CHT and CCVD could be demonstrated only in cases–subjects between baseline and follow-up.

**Figure 3:**
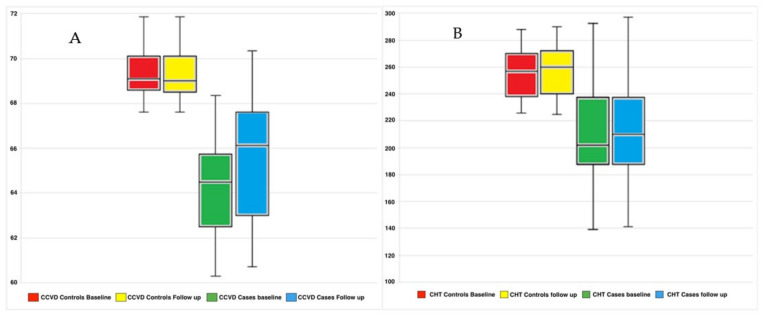
Boxplots showing CCVD (**A**) and CHT (**B**) differences between controls and cases and between cases at baseline and follow-up. Abbreviations: CCVD, choriocapillary vessel density, in percentage; CHT, choroidal thickness, in micron.

**Figure 4:**
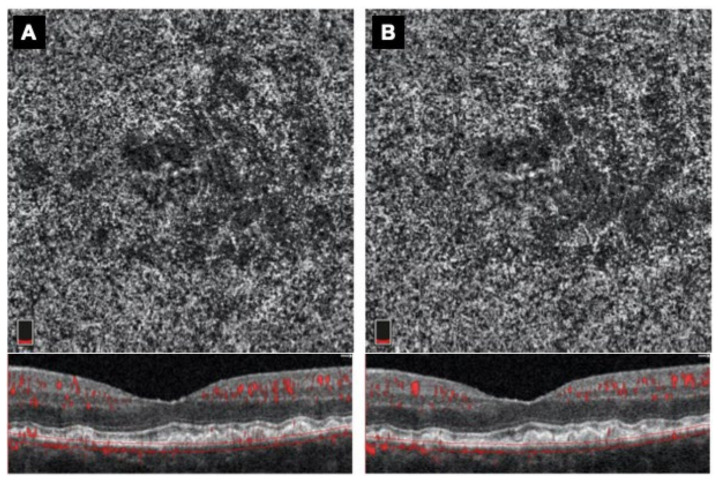
En-face OCTA slab of choriocapillaris and co-registered b-scan with flow overlay (shown in red) at baseline (**A**) and after 6 months (**B**).

On the other hand, the CCVD values showed statistically significant difference between cases and controls at baseline (*p* < 0.001) and in the cases during follow-up after oral therapy (*p* < 0.001, Figure 3A and Figure 4). The CHT measurements showed statistically significant difference between cases and controls (*p* = 0.002) and in the cases during follow-up (*p* < 0.001) (Figure 3B).

**Figure 5:**
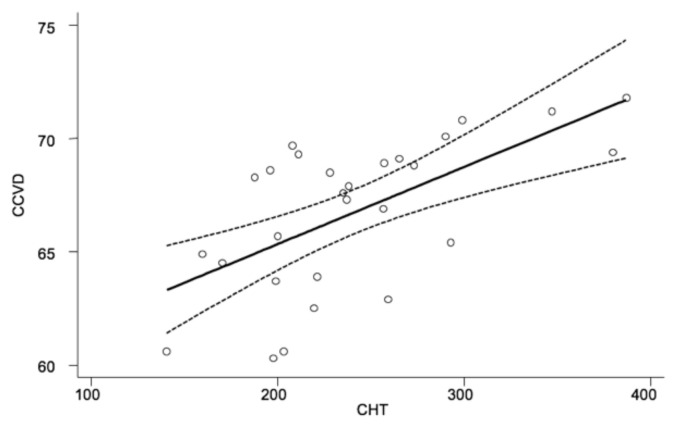
Linear regression analysis evaluating the association between CCVD and CHT in cases and controls. Dotted lines represent 95%CI. R^2^ = 0.39; *p* < 0.001. CCVD (choriocapillary vessel density, in percentage). CHT (choroidal thickness, in micron).

Regarding correlations between clinical variables, significant correlation (r = 0.39; *p* < 0.001) was found between CCVD and CHT at baseline considering both cases and controls (Figure 5).

## 5. Discussion

AMD is still the leading cause of irreversible central vision loss worldwide [2].

Dry AMD is characterized by presence of drusen, pigment abnormalities, and in late stages, by macular GA. It is already known that GA is an area of hypopigmentation where choroidal vessels are more easily visualized because of the degeneration of the retinal pigment epithelium (RPE), followed by dysfunction and death of photoreceptors and choriocapillaris atrophy [19]. It is also known that changes in photoreceptors, retinal pigment epithelium, Bruch’s membrane, and choriocapillaris complex are involved in AMD pathogenesis [5]. Moreover, CC perfusion has a strong association with macular function due to its main role in supplying oxygen to photoreceptors [20,21].

Previous studies have reported a significant reduction in CC perfusion density in eyes suffering from intermediate AMD, especially in concurrence with nascent geographic atrophy or neovascularization in the fellow eye [22]. In wet AMD, CC damage was found to occur in a circumferential zone outside choroidal neovascularization, suggesting primary choriocapillaris loss with a consequent angiogenic signaling from the hypoxic RPE [8].

It has been widely stated that AXT is a form of carotenoid spread in nature with a large variety of physiological and biological activities including antioxidant, anti-tumor, anti-inflammatory, and anti-arteriosclerosis functions [12,23,24,25].

A preliminary human blood rheology study showed a decrease of blood transit time in healthy subjects after AXT ingestion for a ten-day period by collecting blood samples compared with the placebo group [26]. The blood flow improvement after AXT ingestion is likely due to its well-known antioxidant effect on the erythrocyte membranes, thus accelerating blood cell transit.

Our work aimed at investigating blood flow choroidal changes by means of multimodal imaging analysis in patients with intermediate AMD after AXT supplement intake combined with bromelain, vitamin D3, folic acid, lutein, and antioxidants for at least 6 months. In our sample, we excluded eyes with refractive error greater than three diopters because it is known that high myopia affects choroidal thickness and choriocapillaris vessel density [27,28,29].

A previous randomized, double-blind, placebo-controlled study was the first to describe the impact of oral administration of 12 mg AXT on choroidal blood perfusion using Laser speckle flowgraphy in healthy subjects [11].

Conversely, we used both SD-OCT and OCTA imaging for assessing choroidal blood flow changes after AXT supplement intake combined with bromelain, vitamin D3, folic acid, lutein and antioxidants. In particular, we reported the variation of subfoveal choroidal thickness and choriocapillaris vessel density values at baseline and after six months of such combined treatment. Using OCTA, a qualitative and quantitative analysis of retinal and choroidal microvasculature and its changes in ocular diseases are of great interest for both research purpose and clinical practice [8].

In our cohort of patients with AMD, vascular parameters changed significantly during follow-up, likely due to action of oral treatment on choroidal circulation. In detail, a significant increase of both CCVD and CHT was observed, suggesting a potential role of the synergy between AXT, antioxidants, lutein, bromelain, folic acid, and vitamin D_3_ (Astazin 10^®^) on choroidal and choriocapillaris flow improvement.

As reported in the literature, there is a relationship between choriocapillary dysfunction, drusen formation, and AMD progression. Several clinical human studies demonstrated a relationship between CC flow impairment and drusen onset [21]. Moreover, in vivo animal studies on RPE, hypoxia-induced metabolic stress described as hypoxia itself could lead to accumulation of lipid deposits in Bruch membrane within the RPE and in subretinal space, simulating the appearance of early stage of AMD [21]. It has been previously reported that many microorganisms, such as algae, yeasts, and bacilli use AXT as a pigment because it is able to protect them from the ravages of light and stress [12]. Indeed, the xanthophyll group, including AXT, seems to protect human eye lens epithelial cells from UVB-induced stress [27,28]. In addition, AXT has been identified as a suitable multitarget pharmacological agent due to its multiple properties, such as anti-inflammatory, anticancer, antidiabetic, antiapoptotic, and antioxidant activities [30].

In our sample of eyes, a significant correlation between CCVD and CHT at baseline was found in both cases and controls, suggesting a strong connection between these two vascular structures in AMD pathophysiology. To date, choroidal thinning and CC dropout in AMD patients are known, particularly in GA condition.

Oxidative stress and apoptosis of RPE cells leading to their death are key processes in AMD pathogenesis with an evident dysregulation of nutrient exchange pathway between photoreceptors and choriocapillaris [31]. Therefore, AXT, with its effective action to prevent overoxidation of lipids and to control oxidation of LDL [28,31], may prevent RPE cells from oxidative processes and downregulate various inflammatory mediators, thus protecting CC perfusion.

Otherwise, no functional changes in terms of visual acuity were assessed in cases after therapy, although previous studies reported visual acuity and accommodation improvement [27,28].

The main limitation of the study concerns the sample of the study. Our study is a retrospective case series; therefore, it was not possible to assess the sample size and the power of the study. We enrolled all subjects that were available in our dataset. Obviously, the interpretation of the results of the study must be evaluated with caution, keeping in mind that external validity could be questionable, but internal validity was conversely robust.

Other limitations of this study are the following: (i) patients with only intermediate AMD; (ii) the retrospective nature; (iii) structural and functional parameters assessment by a single trained retinal specialist; (iv) scan protocol performed at different daily times, affecting final results of CHT and CCVD measurements due to their circadian fluctuations; (v) the CHT measured only in the subfoveal region.

A sub-analysis of different stages of AMD could be appropriate to validate these results.

## 6. Conclusions

A strength of this study was the detailed analysis of such vasculature structures (choroid and CC), whose quantification remains challenging and questionable.

Further studies with a longer follow-up should be conducted to strengthen these findings and to understand the actual role of AXT assumption, alone or in combination with antioxidants and protective agents, in AMD condition. The accurate segmentation of choriocapillaris is still demanding in patients with different stages of AMD for large drusen or geographic atrophy, confounding flow signal and final analysis.

Moreover, future studies using SS OCTA could help with better visualization of CC with deeper light penetration through RPE.

This study could be considered as a pilot study, useful as a starting point for further perspectives and randomized clinical trials assessing the role of astaxanthin in the treatment of intermediate AMD.

## Figures and Tables

**Table 1:** Demographic and clinical features.

	Baseline			Follow Up	
	Controls	Cases	*p*-Value 1	Cases	*p*-Value 2
Numbers	13	15		15	
Age (yr), mean ± SD	68.92 ± 8.62	70.20 ± 8.13	n.s.	70.20 ± 8.13	n.s.
Gender, n (%)			n.s.		
Male	6 (46)	7 (33)		7 (33)	n.s.
Female	7 (54)	8 (66)		8 (66)	n.s.
Eye, n (%)			n.s.		
R	8 (62)	10 (67)		10 (67)	n.s.
L	5 (38)	5 (33)		5 (33)	n.s.
BCVA (LogMAR)	0.0 ± 0.0	0.1 ± 0.1	n.s.	0.1 ± 0.1	n.s.
IOP (mmHg)	17.42 ± 3.21	16.77 ± 3.85	n.s.	15.65 ± 3.72	n.s.
CHT (micron) CCVD (%)	277.15 ± 61.80 69.42 ± 1.26	210.29 ± 39.81 64.45 ± 7.79	**0.002** **<0.001**	213.65 ± 40.50 65.55 ± 3.13	**<0.001** **<0.001**

*p*-value 1: for the comparison between cases and controls at baseline; *p*-value 2: for the comparison between baseline and follow-up in cases (after treatment). n.s., not significant.

## Data Availability

The datasets used and/or analyzed during the current study are available from the corresponding author on reasonable request.

## Abbreviations

Astaxanthin	AXT
Age-related macular degeneration	AMD
Spectral domain optical coherence tomography	SD-OCT
Optical coherence tomography angiography	OCTA
Retinal pigment epithelium	RPE
Bruch’s membrane	BM
Geographic atrophy	GA
Best-corrected visual acuity	BCVA
Choroidal thickness	CHT
Choriocapillary vessel density	CCVD
Standard deviation	SD

## References

[1] Eye Diseases Prevalence Research Group (2004). Causes and prevalence of visual impairment among adults in the United States. Arch. Ophthalmol..

[2] Coleman H.R., Chan C.C., Ferris F.L., Chew E.Y. (2008). Age-related macular degeneration. Lancet.

[3] Velez-Montoya R., Oliver S.C., Olson J.L., Fine S.L., Quiroz-Mercado H., Mandava N. (2014). Current knowledge and trends in age-related macular degeneration: Genetics, epidemiology, and prevention. Retina.

[4] Ferris F.L., Wilkinson C.P., Bird A., Chakravarthy U., Chew E., Csaky K., Sadda S.R., Beckman Initiative for Macular Research Classification Committee (2013). Clinical classification of age-related macular degeneration. Ophthalmology.

[5] Zarbin M.A., Rosenfeld P.J. (2010). Pathway-based therapies for age-related macular degeneration: An integrated survey of emerging treatment alternatives. Retina.

[6] Wang J.J., Mitchell P., Rochtchina E., Tan A.G., Wong T.Y., Klein R. (2004). Retinal vessel wall signs and the 5 year incidence of age related maculopathy: The Blue Mountains Eye Study. Br. J. Ophthalmol..

[7] Remsch H., Spraul C.W., Lang G.K., Lang G.E. (2000). Changes of retinal capillary blood flow in age-related maculopathy. Graefes Arch. Clin. Exp. Ophthalmol..

[8] Toto L., Borrelli E., Di Antonio L., Carpineto P., Mastropasqua R. (2016). Retinal vascular plexuses’ changes in dry age-related macular degeneration, evaluated by means of optical coherence tomography angiography. Retina.

[9] Boltz A., Luksch A., Wimpissinger B., Maar N., Weigert G., Frantal S., Brannath W., Garhöfer G., Ergun E., Stur M. (2010). Choroidal blood flow and progression of age-related macular degeneration in the fellow eye in patients with unilateral choroidal neovascularization. Investig. Ophthalmol. Vis. Sci..

[10] Feigl B. (2009). Age-related maculopathy—linking aetiology and pathophysiological changes to the ischaemia hypothesis. Prog. Retin. Eye Res..

[11] Saito M., Yoshida K., Saito W., Fujiya A., Ohgami K., Kitaichi N., Tsukahara H., Ishida S., Ohno S. (2012). Astaxanthin increases choroidal blood flow velocity. Graefes Arch. Clin. Exp. Ophthalmol..

[12] Miyawaki H., Takahashi J., Tsukahara H., Takehara I. (2008). Effects of astaxanthin on human blood rheology. J. Clin. Biochem. Nutr..

[13] Mastropasqua R., Di Antonio L., Di Staso S., Agnifili L., Di Gregorio A., Ciancaglini M., Mastropasqua L. (2015). Optical coherence tomography angiography in retinal vascular diseases and choroidal neovascularization. J. Ophthalmol..

[14] Mastropasqua R., D’Aloisio R., De Nicola C., Ferro G., Senatore A., Libertini D., Di Marzio G., Di Nicola M., Di Martino G., Di Antonio L. (2020). Widefield Swept Source OCTA in Retinitis Pigmentosa. Diagnostics.

[15] Mastropasqua R., D’Aloisio R., Di Antonio L., Erroi E., Borrelli E., Evangelista F., D’Onofrio G., Di Nicola M., Di Martino G., Toto L. (2019). Widefield optical coherence tomography angiography in diabetic retinopathy. Acta Diabetol..

[16] Waheed N.K., Moult E.M., Fujimoto J.G., Rosenfeld P.J. (2016). Optical Coherence Tomography Angiography of Dry Age-Related Macular Degeneration. Dev. Ophthalmol..

[17] Tan C.S., Fleckstein M., Schmitz-Valckenberg S., Holz F.G. (2016). Clinical application of multicolor imaging technology. Ophthalmologica.

[18] Samara W.A., Shahlaee A., Sridhar J., Khan M.A., Ho A.C., Hsu J. (2016). Quantitative Optical Coherence Tomography Angiography Features and Visual Function in Eyes with Branch Retinal Vein Occlusion. Am. J. Ophthalmol..

[19] Jia Y., Tan O., Tokayer J., Potsaid B., Wang Y., Liu J.J., Kraus M.F., Subhash H., Fujimoto J.G., Hornegger J. (2012). Split-spectrum amplitude-decorrelation angiography with optical coherence tomography. Opt. Express.

[20] Borrelli E., Uji A., Sarraf D., Sadda S.R. (2017). Alterations in the choriocapillaris in intermediate age-related macular degeneration. Invest. Opthalmol. Vis Sci..

[21] Borrelli E., Mastropasqua R., Senatore A., Palmieri M., Toto L., Sadda S.R., Mastropasqua L. (2018). Impact of Choriocapillaris Flow on Multifocal Electroretinography in Intermediate Age-Related Macular Degeneration Eye. Invest. Ophthalmol. Vis. Sci..

[22] Moult E.M., Waheed N.K., Novais E.A., Choi W., Lee B., Ploner S.B., Cole E.D., Louzada R.N., Lu C.D., Rosenfeld P.J. (2016). Swept-source optical coherence tomography angiography reveals choriocapillaris alterations in eyes with nascent geographic atrophy and drusen-associated geographic atrophy. Retina.

[23] Esterbauer H., Jurgens G., Quehenberger O., Koller E. (1987). Autoxidation of human low density lipoprotein: Loss of polyunsaturated fatty acids and vitamin E and generation of aldehydes. J. Lipid Res..

[24] Li W., Hellsten A., Jacobsson L.S., Blomqvist H.M., Olsson A.G., Yuan X.M. (2004). Alpha-tocopherol and astaxanthin decrease macrophage infiltration, apoptosis and vulnerability in atheroma of hyperlipidaemic rabbits. J. Mol. Cell. Cardiol..

[25] Ohgami K., Shiratori K., Kotake S., Nishida T., Mizuki N., Yazawa K., Ohno S. (2003). Effects of astaxanthin on lipopolysaccharideinduced inflammation in vitro and in vivo. Investig. Ophthalmol. Vis. Sci..

[26] Nakamura A., Isobe A., Otaka Y., Abematsu Y., Nakata D., Honma C., Sakurai S., Shimada Y., Horiguchi M. (2004). Change in visual function from astaxanthin. Jpn. J. Clin. Ophthalmol..

[27] Nitta T., Ohgami K., Shiratori K., Shinmei Y., Chin S., Yoshida K. (2005). Effects of astaxanthin on accommodation and asthenopia—Dose finding study in healthy volunteers. J. Clin. Ther. Med..

[28] Chitchumroonchokchai C., Bomser J.A., Glamm J.E., Failla M.L. (2004). Xanthophylls and alpha-tocopherol decrease UVB-induced lipid peroxidation and stress signaling in human lens epithelial cells. J. Nutr..

[29] Ang M., Wong C.W., Hoang Q.V., Cheung G.C.M., Lee S.Y., Chia A., Saw S.M., Ohno-Matsui K., Schmetterer L. (2019). Imaging in myopia: Potential biomarkers, current challenges and future developments. Br. J. Ophthalmol..

[30] Ambati J., Ambati B.K., Yoo S.H., Ianchulev S., Adamis A.P. (2003). Age-related macular degeneration: Etiology, pathogenesis, and therapeutic strategies. Surv. Ophthalmol..

[31] Giannaccare G., Pellegrini M., Senni C., Bernabei F., Scorcia V., Cicero A.F.G. (2020). Clinical Applications of Astaxanthin in the Treatment of Ocular Diseases: Emerging Insights. Mar. Drugs.

